# Sequential BCR::ABL1 evaluation during dose de-escalation in peripheral blood is more predictive of TFR success than single assessment at dose de-escalation in either peripheral blood or bone marrow

**DOI:** 10.1038/s41375-025-02853-7

**Published:** 2026-01-07

**Authors:** S. D. Patterson, A. Gottschalk, C. Hayden, R. Young, A. Hair, H. G. Jørgensen, H. W. Wheadon, J. F. Apperley, I. Glauche, R. E. Clark, M. Copland, I. Roeder

**Affiliations:** 1 Paul O’Gorman Leukaemia Research Centre, School of Cancer Sciences, College of Medical, Veterinary and Life Sciences, University of Glasgow, Glasgow, UK; 2 Institute for Medical Informatics and Biometry, Faculty of Medicine, Technische Universität Dresden, Dresden, Germany; 3 SIHMDS, North West London Pathology, London, UK; 4 Robertson Centre for Biostatistics, School of Health and Wellbeing, College of Medical, Veterinary and Life Sciences, University of Glasgow, Glasgow, UK; 5 Centre for Haematology, Imperial College London, London, UK; 6 Department of Molecular and Clinical Cancer Medicine, University of Liverpool, Liverpool, UK; 7 National Center for Tumor Diseases (NCT), NCT/UCC Dresden, a partnership between German Cancer Research Center (DKFZ), University Hospital and Faculty of Medicine Carl Gustav Carus TU Dresden, and Helmholtz-Zentrum Dresden-Rossendorf (HZDR), Dresden, Germany

**Keywords:** Translational research, Chronic myeloid leukaemia, Chronic myeloid leukaemia

## Introduction

Continuous tyrosine kinase inhibitor (TKI) treatment provides rapid disease control in almost all patients with chronic phase (CP) chronic myeloid leukemia (CML), leading to near-normal survival [1, 2]. However, most patients report poorer quality-of-life due to TKI-related side-effects [3–6]. Some of the CP-CML patients achieving deep molecular responses (DMR; BCR::ABL1^IS^$\,\le$ 0.01%) can stop their TKI, often after many years of therapy, and successfully maintain major molecular response (MMR; BCR::ABL1^IS^ $\le$0.1%), termed “treatment-free remission” (TFR) [7]. Longer durations of TKI treatment and DMR are associated with improved rates of successful TFR; however, a reliable prediction of individual TFR success is not possible.

In the DESTINY trial [8] the TKI dose was reduced by 50% for 12 months followed by TKI cessation for eligible CP-CML patients (Supplementary Information). This de-escalation approach reduced TKI-related side-effects and costs whilst maintaining efficacy, and further demonstrated that BCR::ABL1^IS^ kinetics in peripheral blood (PB) during TKI de-escalation are predictive for molecular recurrence after TKI stop [9].

Here, we aimed to determine if the evaluation of *BCR::ABL1* in bone marrow (BM) mononuclear cells (MNCs), which contain the primitive CML stem/progenitor cells, at the point of TKI de-escalation is more predictive of TFR than PB evaluation of total leukocytes, and whether existing prediction models could be improved by combining PB and BM measurements. Specifically, we investigated if the use of repeated PB evaluations during TKI dose de-escalation can add predictive power to the assessment of BCR::ABL1^IS^ levels at the time of TKI dose de-escalation.

## Materials and methods

MNCs or total leukocytes were isolated from BM and PB samples, respectively, taken immediately prior to TKI de-escalation (timepoint ‘0’). Of the patients enrolled in DESTINY (*n* = 174), we conducted paired analyses of PB and BM on a subset using available samples (*n* = 107), in which BCR::ABL1^IS^ was determined by qRT-PCR (see Supplementary Fig. 1, Supplementary Tables 1&2, Supplementary Information).

During TKI de-escalation, the molecular response was monitored monthly in PB. Linear regression modeling was applied to describe changes in PB BCR::ABL^IS^ during TKI de-escalation [9]. Using a minimum of three sequential detectable BCR::ABL1^IS^ values, regression parameters, i.e., slope and intercept, were individually determined for all eligible DESTINY patients. In addition, BCR::ABL1^IS^ values from sequential PB samples during TKI de-escalation were compared with single BCR::ABL1^IS^ measurements taken prior to TKI de-escalation from either BM MNCs or PB total leukocytes (Supplementary Fig. 2).

## Results

**Fig. 1:**
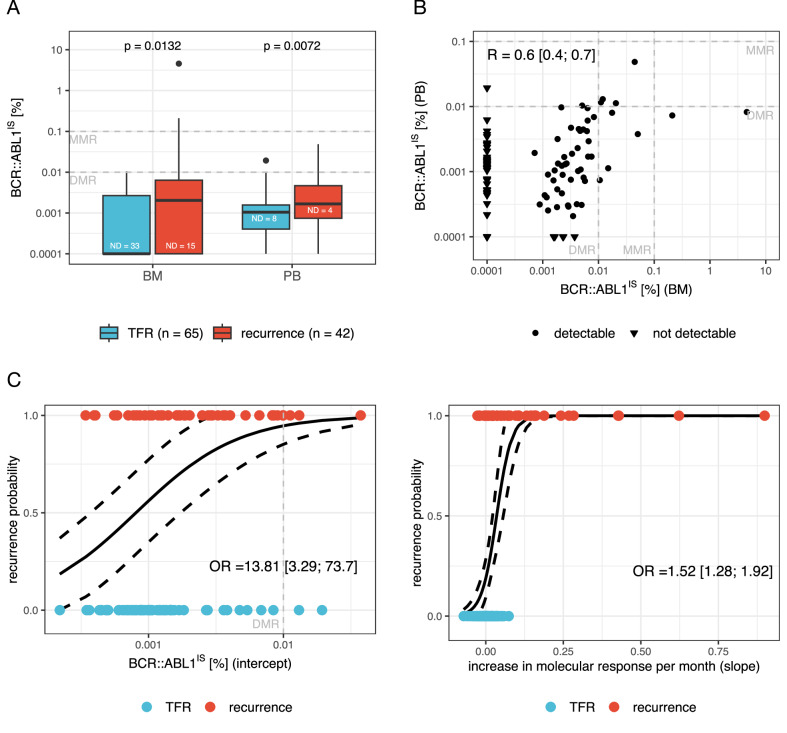
BCR::ABL1^IS^ measurement from peripheral blood (PB) leukocytes is more predictive of TFR outcome than from bone marrow (BM) mononuclear cells (MNCs). **A** Box plots showing the median (black line) and interquartile range (box) for molecular response levels (c.f. Supplementary Information) detected in BM MNCs (TFR (blue): median = −4 with IQR = [-4; -2.57], recurrence (red): median = −2.69, IQR = [-4; -2.2]) and PB leukocytes (TFR (blue): median = −2.98 with IQR = [-3.39; -2.81], recurrence (red): median = −2.78, IQR = [-3.13; -2.33]; all given as log-reduction, i.e. log_10_[*BCR::ABL1*^*IS*^]). Samples for which the molecular response was below the limit of detection are denoted as ‘not detectable’ (ND). *p*-values are derived from Mann–Whitney U-tests, comparing the median molecular response between TFR and recurrence patients for BM MNC and PB leukocyte measurements, respectively. **B** Molecular response values as detected in BM MNCs and PB leukocytes immediately prior to dose de-escalation. For correlation analysis, ND values were excluded and the Spearman’s correlation coefficient (R) is shown with the associated 95% CI. *p* < 0.001 for testing the null hypothesis R = 0. **C** Recurrence probabilities estimated by multivariate logistic regression analyses (solid black line, dashed black line: 95% CI) from sequential PB leukocyte measurements during dose de-escalation using the calculated intercept (left) and slope (right) as independent explanatory variables. Dots are individual intercept (left) and slope (right) values of patients who showed TFR (blue) or recurrence (red). The intercept is given in terms of molecular response and the slope in terms of increase in molecular response per month. Estimated ORs and associated 95% CIs describe the increase in the chance of losing TFR if intercept is increasing by one unit and if slope is increasing by 0.01 unit. BM bone marrow, PB peripheral blood, ND not detectable, TFR treatment-free remission, MNC mononuclear cell, IQR interquartile range, CI confidence interval, OR odds ratio, MMR major molecular response, DMR deep molecular response.

Patient demographics and selected clinical parameters are shown in Supplementary Table 1. In this cohort, 92 patients were treated with imatinib, 9 with nilotinib and 6 with dasatinib. The median BCR::ABL1^IS^ value at trial entry was significantly higher in both BM and PB in patients who experienced recurrence (Fig. 1A). However, BCR::ABL1^IS^ was not detectable in the BM, but detectable in PB for 42 patients (39.3%), while it was undetectable in both BM and PB for 6 patients (5.6%) or undetectable in PB and detectable in BM for 6 patients (5.6%), suggesting PB sampling may be more sensitive (Fig. 1B). There was neither a significant difference in the BM aspirate white cell counts with respect to detectability of BCR::ABL1 nor a significant association between detectability and MNCs used as input for RNA isolation and RT-qPCR preparation (Supplementary Fig. 3).

Using only samples with detectable BCR::ABL1^IS^ values (*n* = 53; 49.5%), transcript levels were moderately correlated between BM MNCs and PB leukocytes (Fig. 1B). Furthermore, we observed a statistically significant trend, with lower median BCR::ABL1^IS^ levels at trial entry associated with longer times to recurrence, in both BM MNCs and PB leukocytes (Supplementary Fig. 4). This indicates that higher BM MNC BCR::ABL1^IS^ levels prior to dose de-escalation are associated with a shorter time to relapse, in parallel with previous findings based on PB leukocyte BCR::ABL1^IS^ [10].

Univariate logistic regression shows that BCR::ABL1^IS^ levels, from either BM MNCs or PB leukocytes taken at TKI de-escalation, were similarly predictive of TFR success at 36 months post-de-escalation (Supplementary Figs. 5A, B). Thus, a benefit of BM MNCs over PB leukocyte evaluations at the point of de-escalation was not shown.

Next, we used sequential PB BCR::ABL1^IS^ levels taken during the 12 months TKI de-escalation to fit a linear regression model. The estimated regression parameters, intercept and slope, were tested as predictors of TFR success. In the univariate model, the intercept was associated with a higher odds ratio (OR) compared to a single BCR::ABL1^IS^ value from BM MNCs or PB leukocytes at trial entry, as previously determined (Supplementary Fig. 5C). In contrast, the regression slope did not show an increased OR, compared to the single BM MNC or PB leukocyte values at de-escalation (Supplementary Fig. 5D). However, when using both the regression slope and intercept as independent predictors in a multivariate logistic regression model, the predictive value of the intercept was even more pronounced if adjusted for the slope effect, which itself remained predictive of TFR (Fig. 1C, Supplementary Table 3).

**Fig. 2:**
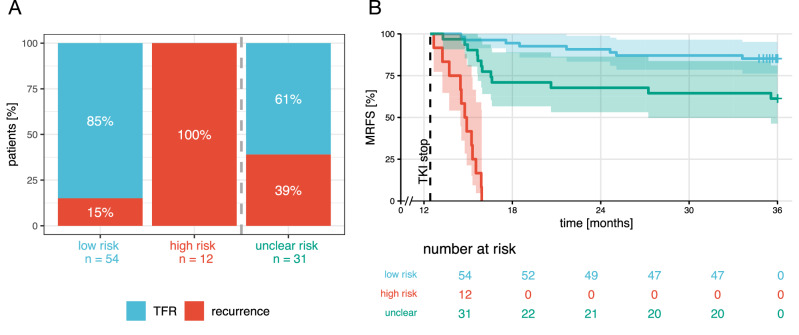
Risk of relapse following TKI de-escalation can be classified based on intercept and slope, estimated from sequential peripheral blood-based BCR::ABL1^IS^ measurements during TKI dose de-escalation. **A** Proportion of patients in TFR or with recurrence events until the end of the 24-month follow-up period after TKI cessation in the three risk groups based on joined classification using intercept and slope. Absolute patient numbers given below the bars. **B** Kaplan–Meier curve for MRFS (shaded areas: 95% confidence intervals), stratified according to the three risk classification groups; low, high and unclear. The dashed line represents the end of the de-escalation phase (12 months). The trial endpoint was set to 36 months, i.e., 24-month follow-up post-TKI cessation. Thus, patients were censored at last seen date before or at the end of trial. TFR Treatment-free remission, MRFS molecular recurrence-free survival, TKI tyrosine kinase inhibitor.

Based on these findings, we used the slope and intercept to derive a risk prediction model for molecular recurrence. Using a comparative ROC analysis (Supplementary Information), we classified DESTINY patients into three risk groups: *low*, *high* and *unclear* (Fig. 2, Supplementary Fig. 6A, B). For patients who could be classified into the *high-* or *low-risk* group, this model had high positive (PPV) and negative predictive values (NPV) of 100% and 85.2%, respectively, and a misclassification rate of only 12.1%. The difference in molecular recurrence-free survival between the risk groups is illustrated in Fig. 2B using stratified Kaplan–Meier curves.

In comparison to the slope and intercept evaluation, risk classification using single-timepoint BCR::ABL1^IS^ measurements from either BM MNCs or PB leukocytes immediately prior to de-escalation were not as predictive of molecular recurrence, regarding both PPV and NPV. Also, both models had higher misclassification rates (Supplementary Fig. 7). Furthermore, adding these single-timepoint BM MNC or PB leukocyte measurements to the multivariate (intercept + slope) model as independent predictors did not result in a better model fit (Supplementary Table 4).

## Discussion

Our results show that there is no benefit from quantifying BCR::ABL1^IS^ from BM MNCs over PB leukocytes for the prediction of TFR success, an important conclusion for patients. Furthermore, we demonstrate that TFR success can be more reliably predicted using the BCR::ABL1^IS^ slope and intercept estimated by a linear regression from sequential PB evaluations during TKI de-escalation compared to single BM MNC or PB leukocyte-derived values determined prior to de-escalation. This approach was limited by access to at least three quantifiable BCR::ABL1^IS^ measurements which, however, were available for >99% of patients in the DESTINY cohort.

Although the robustness of the estimated cut-off values for slope and intercept has been shown by cross-validation analysis (Supplementary Information), we emphasize that the proposed classification scheme needs to be validated in independent patient cohorts. Nevertheless, the presented results already highlight the additional information provided by BCR::ABL1^IS^ kinetics during TKI de-escalation. Specifically, we showed that whenever ≥3 PB BCR::ABL1^IS^ measurements are available, the BCR::ABL1^IS^ at the time of TKI de-escalation can more reliably be estimated by the regression intercept than by a single evaluation of either PB leukocyte or BM MNC samples.

Beyond TFR prediction, we observed that for 8 of 10 patients who experienced recurrence during TKI de-escalation, a *high-risk* classification was evident; the remaining two patients were classified as *unclear* (Supplementary Fig. 8). This suggests that molecular recurrence risk may be predicted based on the first 3 months of TKI de-escalation (Supplementary Fig. 9). However, to validate this finding and to identify the shortest necessary monitoring period with the same predictive power as the entire 12 months of de-escalation, more independent patient data would be required.

In our analysis, there were very few undetectable BCR::ABL1^IS^ values from PB during TKI de-escalation. Thus, these could be omitted without affecting the results, and estimation of the regression slope and intercept is possible from ≥3 PB BCR::ABL1^IS^ measurements for almost all patients. To estimate these parameters for patients with undetectable BCR::ABL1^IS^ values, more sophisticated methods would be necessary. Undetectable BCR::ABL1^IS^ values also raise another important issue regarding the comparison of PB leukocytes versus BM MNCs. In 42 patients, BCR::ABL1^IS^ was detectable in PB leukocytes, but not BM MNCs. A possible reason for this is that patients in DMR may only have scattered small pockets, if any, of *BCR::ABL1* positive cells within the BM, as suggested by imaging results for murine progenitor cells [11]. Therefore, obtaining a positive *BCR::ABL1* result is dependent on the area of the BM being sampled, whereas PB is assumed to provide a more homogenous *BCR::ABL1* signal. This observation supports the use of sequential PB-based BCR::ABL1^IS^ measurements as a more robust method to predict TFR success.

Within DESTINY, late molecular recurrences occurred beyond the trial endpoint of 24 months post-TKI cessation for *n* = 5 patients (Supplementary Fig. 10). In the presented analyses, these patients’ outcomes are classified as TFR, as recorded at the endpoint. Of these 5 patients, 3 were classified as *low-risk*, and their BM and PB BCR::ABL1^IS^ values at study entry were relatively low and comparable to those of the other TFR patients. If these patients were analyzed as recurrences, the classification cut-off value for the regression slope would change considerably: while the misclassification rate among *high*/*low*-*risk* patients would decrease from 12.1% to 7.7%, the number of patients with *unclear-risk* would increase from 32.0% to 46.4%. This illustrates that the classification is dependent on the timing of recurrence. Accordingly, if the BCR::ABL1^IS^ slope remains positive, molecular recurrence will eventually occur; however, the timing of the recurrence depends quantitatively on the slope as well as on the intercept, which is effectively a robust estimate of the molecular response at the time of TKI de-escalation.

In conclusion, our results provide strong evidence that PB leukocyte BCR::ABL1^IS^ kinetics during TKI dose de-escalation add considerable prognostic power for TFR success and BCR::ABL1^IS^ evaluation from BM MNCs at the time of TKI de-escalation does not add prognostic information. Implementing the proposed classification could improve TFR rates for *low-risk* patients while minimizing the risk of molecular recurrence for *high-risk* patients. Specifically, the de-escalation phase could be used to determine if or when a patient could discontinue TKI successfully. However, to validate the suggested cut-off values, independent data on patients with altered TKI dose before treatment stop is necessary. Thereafter, a prospective clinical trial should test the suggested three-group risk classification in terms of TFR success. Furthermore, given that most patients in this cohort were treated with imatinib prior to TKI de-escalation, our observations are less generalizable to CML patients treated with other TKIs, which might be associated with distinct BCR::ABL1 kinetics. As such, validation of our classification model in a subsequent clinical trial should also include a comparative analysis of patients treated with later-generation TKIs, thereby supporting wider clinical translation of this study’s findings.

## Supplementary information

Supplemental Materials

## Data Availability

All data generated or analysed using PB-derived samples during this study are available as published in [9]. Researchers wishing access to the BM qRT-PCR data should contact the corresponding author.
