# Supplementary material for: Sequential BCR::ABL1 evaluation during dose de-escalation in peripheral blood is more predictive of TFR success than single assessment at dose de-escalation in either peripheral blood or bone marrow
Source: Leukemia. 2026 Jan 7;40(2):459–63. doi: 10.1038/s41375-025-02853-7 (PMC12875873; doi:10.1038/s41375-025-02853-7)
Supplement: Supplementary file 1 — Supplemental Materials [file 41375_2025_2853_MOESM1_ESM.docx]

**Supplemental Information Contents**

*(.pdf file)*

- **Clinical trial data:** Details of the DESTINY study and patient cohort.
- **Methods to measure BCR::ABL1^IS^:** Technical description of methods of BCR::ABL1^IS^ quantification.
- **Statistical methods:** Description of applied statistical analyses procedures including results (e.g., model parameters).
- **Supplemental Figures [1–11] and Tables [1–9].**
- **Supplemental references [S1-S5].**

**Clinical trial data**

All presented analyses are based on data from the DESTINY trial (NCT01804985; [S1, S2]). In this trial, each patient’s TKI dose was reduced by 50% of the respective standard dose for 12 months (de-escalation phase) followed by TKI cessation (stop phase). In total n=174 chronic phase CML patients in stable remission were included in the trial. Of these, n=125 were in deep molecular remission (DMR) and n=49 in major molecular remission (MMR); n=148 were treated with imatinib, n=16 with nilotinib, and n=10 with dasatinib.

Clinical BCR::ABL1^IS^ transcript data from the peripheral blood (PB) of DESTINY trial participants (n=174) were available at the point of trial entry immediately prior to de-escalation (as published in [S3]) and sequentially following this period. Additionally, mononuclear cells (MNCs) were isolated from bone marrow (BM) and total leukocytes were isolated from PB samples taken immediately prior to TKI de-escalation. n=107 patients could be included in the final analyses (see Suppl. Fig. 1, Suppl. Tab. 1).

Patient outcomes were determined 24 months post-TKI cessation as either ‘TFR’ (treatment-free remission) or ‘recurrence’ (molecular recurrence). At 24 months post-TKI cessation, the rates of molecular recurrence-free survival (MRFS) were 72% and 36% in the DMR and MMR groups, respectively. According to the DESTINY study protocol [S1, S2], molecular recurrence was defined as the first of two consecutive measurements reporting BCR-ABL1^IS^ >0.1%. Furthermore, we considered n=3 additional recurrences reported in *case report forms,* in which the BCR-ABL1^IS^ >0.1% threshold was not reached. This corresponds to final trial results posted on EUDRACT.

**Methods to measure BCR::ABL1^IS^**

Sample Processing and Cell Lysate Preparation

PB white blood cell lysates were prepared by selective lysis of red blood cells using the Puregene RBC Lysis Solution (Qiagen), followed by washing with phosphate-buffered saline (PBS) (Thermo Fisher Scientific). BM samples were processed to isolate MNCs, which were subsequently preserved in RNAlater (Thermo Fisher Scientific) at –20°C. Prior to lysis, RNAlater was removed, and a lysis buffer consisting of Buffer RLT Plus (Qiagen), 2-mercaptoethanol (Sigma-Aldrich), and Reagent DX (Qiagen) was added to both PB and BM cell pellets. Homogenization was carried out using the TissueLyser II (Qiagen), and the resulting cell lysates were stored at –20°C until further processing.

RNA Extraction and cDNA Synthesis

Total RNA was extracted from PB and BM lysates using the QIAsymphony platform in conjunction with the QIAsymphony RNA Kit (Qiagen). Complementary DNA (cDNA) was synthesised from total RNA using the LunaScript™ RT SuperMix Kit (New England Biolabs), following the standard reverse transcription protocol.

Real-Time Quantitative PCR (RT-qPCR)

RT-qPCR was performed on the QuantStudio™ 5 Real-Time PCR System (Thermo Fisher Scientific) using a duplex assay targeting the *BCR::ABL1* fusion transcript (e13a2/e14a2) and the *ABL1* reference gene. Custom TaqMan™ primers and probes (Thermo Fisher Scientific; see Suppl. Tab. 2) were used in conjunction with the TaqMan™ Fast Advanced Master Mix (Thermo Fisher Scientific). Quantification was calibrated using the ERMAD623 *BCR-ABL* plasmid DNA (ERM®).

PCR reactions (20 µL total volume) contained 3 µL of cDNA and were run in triplicate under the following cycling conditions: polymerase activation at 95°C for 20 seconds, followed by 45 cycles of denaturation at 95°C for 3 seconds and annealing/extension at 60°C for 45 seconds (fast-mode). Results were expressed as *BCR::ABL1*/*ABL1* ratios and normalised to the international scale (IS) using a validated laboratory-specific conversion factor. A minimum of 10,000 *ABL1* copies was required for reliable *BCR::ABL1* quantification. Data analysis was performed using QuantStudio™ Design and Analysis Software v1.5.1 (Thermo Fisher Scientific).

**Statistical methods**

*Molecular response evaluation*

To describe and model the BCR::ABL1^IS^, we applied the *BCR::ABL1* to *ABL1* ratio converted to the international scale (*IS*), i.e., (*BCR::ABL1 / ABL1)* $\cdot$ *100%* $\cdot$ *CF* = BCR::ABL1^IS^, with the conversion factor of the particular lab (*CF*). Molecular response (MR) levels are given in terms of log reductions: log_10_[(*BCR::ABL1 / ABL1)* $\cdot$ *CF*] = log_10_[*BCR::ABL1 ^IS^*] (e.g. *BCR::ABL1 ^IS^ = 0.001 which equals log_10_[BCR::ABL1 ^IS^] = -3 which is denoted as MR3, i.e. 3-fold log-reduction).* Values below MR6 (limit of detection, LOD), i.e. BCR::ABL1^IS^ $\leq$ 0.0001%, are denoted as *undetectable*. Undetectable measurements are only considered for the statistical analysis of BM MNCs and PB leukocytes prior to TKI dose de-escalation. After TKI dose de-escalation, BCR::ABL1^IS^ below the detection limit in PB are not considered for the statistical analysis [S3]. Please note that all analyses that include such undetectable values are highly dependent on the chosen LOD cut-off, particularly if it exceeds the smallest measurable value. In our analysis, the LOD of MR6 is below the smallest measurable value and receives the lowest rank of all measurements. Therefore, all rank-based analyses are not dependent on this.

*Statistical analyses methods to analyse molecular response levels*

Using Mann–Whitney U-tests we compared the median molecular response between ‘TFR’ and ‘recurrence’ patients for BM and PB measurements, respectively (Fig. 1 A). To statistically test the trends of molecular responses (i.e. null hypothesis: equality of all three groups) we applied Kruskal–Wallis tests (Suppl. Fig. 4).

For correlation analysis of molecular response levels (Fig. 1B), undetectable values were excluded and the Spearman’s correlation coefficient (R) with the associated 95% CI was estimated. The p-value is given for testing the null hypothesis: R=0.

*Linear regression for parameter estimation during TKI dose de-escalation (see also* [S3])

Time courses of the log_10_[BCR::ABL1^IS^] values (denoted as $LRATIO(t)$) within the 12 months dose reduction period are described by a linear function depending on time 𝑡 (in months), i.e.: 𝐿𝑅𝐴𝑇𝐼𝑂(𝑡) = 𝑏 + 𝛽𝑡 with intercept parameter 𝑏 and slope parameter 𝛽, assuming independent, normally distributed errors with constant variance 𝜎2. The independence assumption is a simplification. We, therefore, tested for a potential autocorrelation effect within the BCR::ABL1^IS^ time courses and the results of this analysis substantiated our simplifying assumption (see section “*Testing for auto-correlation in BCR::ABL1^IS^ time courses” below).* Thus, for each individual patient 𝑖, we applied a standard linear regression model (R-package: *stats*, function: *lm*()) to fit the $LRATIO(t$) function, thereby obtaining estimates of the intercept $\hat{b}_{i}$ and the slope$\hat{\beta}_{i}$along with an estimated residual variance $\hat{\sigma}_{i}^{2}$ on the log-scale. The fitting routine is only applied if at least three eligible observations are obtained during the dose reduction period. An observation is considered eligible when: 1) BCR::ABL1^IS^ is detected (i.e. measurements are above the individual detection limit of the qRT-PCR) and 2) it occurred before a confirmed molecular recurrence (cited from [S3]). Finally, we included n=107 patients in the analysis, which fulfil those criteria. The median number of used samples per patient was 11 (IQR: 9–12; range: 3–16). To check for the sample size effect on the precision of the estimates, we examined the correlation between the number of samples per patient and the estimated standard errors for the intercept and slope obtained from the fitting routine. For the intercept, no association was observed, whereas for the slope, only a weak negative correlation was found using Spearman’s correlation coefficient (Suppl. Fig. 11), demonstrating that the individual parameter estimates are comparable.

*Logistic Regression*

A logistic regression model was applied to analyze whether, besides the change of the BCR::ABL1^IS^ values during the 12 months TKI dose de-escalation period (slope $\beta$), the estimated intercept *b* or the log_10_[BCR::ABL1^IS^] level prior TKI dose de-escalation period *c* (from BM) and *d* (from PB) are predictive for recurrence at any time after trial entry. Technically, we transformed the individual slope parameter 𝛽′ = 𝛽 ∙ 100 to achieve better numerical robustness. In the **full models** we analyzed at maximum three predictors for the probability 𝜋 of the occurrence of molecular recurrence (R-package: *stats*, function: *glm*()). I.e., given the probability P(recurrence | 𝛽´, *b*, *c*) = 𝜋(𝛽´, *b*, *c*), the logistic regression model is

$$\ln\left( \frac{\pi\left( \beta´, b, c \right)}{1-\pi\left( \beta´, b, c \right)} \right)= \gamma_{0}+\gamma_{1}\beta´+\gamma_{2}b+\gamma_{3}c$$

and given the probability P(recurrence | 𝛽´, *b*, *d*) = 𝜋(𝛽´, *b*, *d*), it is

$$\ln\left( \frac{\pi\left( \beta´, b, d \right)}{1-\pi\left( \beta´, b, d \right)} \right)= \gamma_{0}+\gamma_{1}\beta´+\gamma_{2}b+\gamma_{4}d$$

with offset $\gamma_{0}$ and parameters $\gamma_{i}$, $i=\left\{ 1, 2, 3,4 \right\}$.

We applied Wald tests and profile likelihood confidence intervals to assess the statistical significance of the predictor estimates.

*Model comparison and parameter selection*

All model fits and model characteristics are summarized in Suppl. Tabs. 3 and 4.

In the first step, we applied univariate logistic regression with only one of the four variables ($\beta´, b, c,d$) as predictors and compared these four models with the Akaike information criterion (AIC), looking for the most suitable model (i.e. with lowest AIC). The AIC is given by AIC = 2k−2log(L), with k as the number of estimated parameters and L as the maximum likelihood of the particular model. The model with the slope $\beta´$ showed the lowest AIC (similar to [S3]).

In the second step, we asked whether the individually estimated intercept improved the univariate model, and compared the univariate model of predictor slope $\beta´$ with the bivariate model with the predictors slope $\beta´$ and intercept *b.* Using the likelihood ratio test (LRT), we compared the two nested models (R-package: *stats*, function: *anova*()). The test statistic is given by D=−2⋅(logL_0_−logL_1_), with L_0_ = likelihood of the smaller model and L_1_ = likelihood of the more complex model. D∼χ2(df), where df is the number of additional parameters in the more complex model. The individually estimated intercepts *b* improved the univariate model and we used the bivariate model as the reference model for the following steps.

In the third step, we asked whether molecular response levels prior TKI dose de-escalation period *c* from BM or *d* from PB improve the bivariate model. Therefore, we again used LRT to compare the nested models and could show that there is neither further improvement by molecular response levels prior TKI dose de-escalation from BM nor from PB. Thus, the most predictive model is the bivariate model

$\ln\left( \frac{\pi\left( \beta´, b \right)}{1-\pi\left( \beta´, b \right)} \right)= \gamma_{0}+\gamma_{1}\beta´+\gamma_{2}b$.

*Identification of different response groups using ROC classification*

Due to the finding that the variables slope 𝛽 during dose de-escalation and intercept *b*, as well as molecular response level prior to TKI dose de-escalation from BM $c$or PB $d$ are predictive as univariate predictors for the occurrence of molecular recurrence at any time after trial entry, we aimed to categorize the patients into clinically relevant groups. In order to identify a cut-off value that adequately separates the patients into risk groups, we used receiver operating characteristics (ROC). The four predictors ($\beta´, b, c,d$) were studied with respect to their ability to predict the molecular recurrence by analysing the cut-off-specific sensitivities and specificities in a ROC curve (Suppl. Fig. 6 for $\beta´, b$). Herein the sensitivity and specificity are given as 𝑠𝑒𝑛𝑠 = 𝑡𝑝/(𝑡𝑝 + 𝑓𝑛) and 𝑠𝑝𝑒𝑐 = 𝑡𝑛/(𝑡𝑛 + 𝑓𝑝), respectively, with 𝑡𝑝 = true positive, 𝑡𝑛 = true negative, 𝑓𝑝 = false positive and 𝑓𝑛 = false negative. From this, we can derive the maximum Youden-Index [S4], which maximizes the sum of sensitivity and specificity (Youden-Index: 𝐽 = 𝑠𝑒𝑛𝑠 + 𝑠𝑝𝑒𝑐 − 1) as one possible cut-off value**.**

To assess the classification quality of the different parameters, we calculate the classification error as well as the positive (PPV) and negative (NPV) predictive value, the latter referring to the probability of recurrence in the *high*-*risk* group and the probability of TFR in the *low-risk* group, respectively (Suppl. Tab. 5). Mathematically, the classification error is defined as *cr* = ((f𝑝 + f𝑛)⁄(𝑡𝑝 + 𝑡𝑛 + 𝑓𝑝 + 𝑓𝑛)) and the 95% confidence interval $cr\pm1.96\cdot\sqrt{cr\cdot(1-cr)/(tp + tn + fp + fn})$, while the positive predictive value is given as 𝑝𝑝𝑣 = 𝑡𝑝⁄𝑡𝑝 + 𝑓𝑝] with 95% confidence interval $ppv \pm1.96\cdot\sqrt{{ppv \left( 1 - ppv \right)}/{(tp + fp)}}$ and the negative predictive value is given as 𝑛𝑝𝑣 = 𝑡𝑛/(𝑡𝑛 + 𝑓𝑛)] with 95% confidence interval $npv \pm1.96\cdot\sqrt{{npv \left( 1 - npv \right)}/{(tn + fn)}}$.

Based on the classification, we estimated the odds ratios 𝑂𝑅=(𝑡𝑛∙𝑡𝑝)/(𝑓𝑛∙𝑓𝑝) and the 95% confidence interval $95\%CI=OR\cdot\text{exp}\left( \pm1.96\cdot\sqrt{\frac{1}{tn}+\frac{1}{tp}+\frac{1}{fn}+\frac{1}{fp}} \right)$, indicating whether the risk of recurrence is increased for the *high*-*risk* group compared to the *low-risk* group (Suppl. Tab. 6).

According to the reference logistic model, which is most predictive for TFR success (i.e. with predictors slope and intercept), we further developed an extended risk classification using three risk groups: *low-risk, high-risk, unclear risk.* Patients with both low intercept and negative or low slope value (i.e. intercept < -2.8 and slope < 0.035) were classified as *low-risk* (n=54, 50%), whereas patients with both high intercept and high slope value (i.e. high intercept > -2.8 and high slope > 0.035) were classified as *high-risk* (n=20, 19%). The remaining patients with either high intercept or high slope value (i.e. intercept > -2.8 and slope < 0.035 or intercept < -2.8 and slope > 0.035) were separated into *unclear risk* (n=33, 31%) (Suppl. Tabs. 5 and 6).

This 3-group classification is just one option, aiming for trustworthy statements with respect to TFR prediction. Although providing very low misclassifications of *high-* and *low-risk* predictions, it comes at the cost of a cohort of patients with unclear risk. Note that the model predictions are highly dependent on how the cut-off values are chosen and which optimization criterion is used. This choice depends on the primary goal: minimizing false TFR prediction or minimizing false recurrence predictions. Whereas the described 3-group-classification balances sensitivity (avoiding failure to predict TFR-loss) and specificity (avoiding prediction of recurrence for TFR patients), it would also be possible to maximize either sensitivity or specificity alone, but at the expense of each another.

*Cross-validation of the cut-off values*

To evaluate the (internal) robustness of the cut-off values of the intercept (–2.8) and slope (0.035) obtained from the ROC analysis, we applied different cross-validation (CV) methods, including leave-one-out CV (LOOCV), leave-10%-out CV, and 5-fold CV. To compare the original classification method with the CV approaches, we examined the number of patients classified as unclear risk and the number of misclassifications. The CV methods differ only in the composition of the training and test sets. The training set was used to calculate the cut-off values by ROC analysis. Based on these values, risk classification of the patients in the test set was performed as described above.

In LOOCV, all but one patient, who represents the test set, are used in the ROC analysis as the training set to determine the cut-off values. This results in a predicted classification for every patient, which can then be compared with its observed molecular outcome. For the 10%-out CV, 10% of the patients were randomly selected as the test set, while the remaining 90% of the patients were used as training set for cut-off calculation. This procedure was repeated up to 1,000 times to obtain a distribution of the number of unclear-risk patients and the classification error. For the 5-fold CV, patients were divided into k = 5 blocks. Each block was used once as the test set, with the remaining blocks serving as the training set. In total, the validation was repeated 5 times, yielding 5 estimates of the number of unclear-risk patients and 5 classification errors. These estimates were then averaged to obtain the median values.

The results of the cross-validation studies (Suppl. Tab. 7) demonstrate internal robustness of the estimation procedure. However, independent patient cohorts are necessary to check for external validation.

*Time to molecular recurrence*

In order to analyse time to event data, we estimate the molecular recurrence-free survival (MRFS) probability after TKI discontinuation (Fig. 2B) by a Kaplan-Meier estimator (R-package: *survival*). We stratified according to the three risk groups *low-risk*, *high-risk* and *unclear-risk* based on the cut-off values for the individual slope and intercept parameter. Patients with molecular recurrence during TKI dose de-escalation (n=10) were excluded for this survival analysis. See section “Risk classification based on the dynamics of 3 months under TKI dose de-escalation” for prediction of recurrence events during de-escalation phase.

*Testing for auto-correlation in BCR::ABL1^IS^ time courses*

Time-course data are potentially autocorrelated, meaning that a given time point is influenced by preceding time points. For the sake of simplicity and clinical feasibility, we did not account for such a dependency structure in addition to the slope parameter in our linear regression analysis (see section “*Linear regression for parameter estimation during TKI dose de-escalation” above)*. To substantiate this simplifying approach, we applied a population-based linear mixed-effects model for estimating $LRATIO(t)$ within the 12 months dose de-escalation period:

First, both intercept and slope were estimated as fixed and random effects to account for patient heterogeneity without considering autocorrelation:

$$LRATIO_{i}(t) = {{(\omega}_{b}}_{i}+b) + {{(\omega}_{\beta}}_{i}+\beta)\cdot t$$

where ${\omega_{b}}_{i}$ and ${\omega_{\beta}}_{i}$denote the patient-specific random effects for the intercept and slope, respectively, and 𝑏 and 𝛽 represent the corresponding fixed effects, under the assumption of normally distributed errors with constant variance 𝜎2. We applied maximum likelihood (ML) estimation (R-package: *nlme*, function: *lme*()) to fit the $LRATIO(t$) function, thereby obtaining estimates of the intercept $\hat{b}$ and the slope$\hat{\beta}$along with its standard deviation $\hat{\omega}_{\beta}$ and $\hat{\omega}_{b}$ and an estimated residual standard deviation $\hat{\sigma}$ on the log-scale.

Second, we fitted an extended model, in which an autocorrelation parameter $\phi$ for a continuous autoregressive process of order 1 is estimated. In this model, the patient-specific residual $e_{ij}$ at time $t_{ij}$ is correlated with the residual $e_{ik}$ at a previous time $t_{ik}$, taking the actual time difference into account: $Corr(e_{ij},e_{ik})=exp(-\phi\cdot|t_{ij}-t_{ik}|)$. Here, $\phi>0.$The larger $\phi$ is, the faster the correlation decays with increasing time distance.

The results of the basic (reference) and the extended model, based on n=107 patient time courses, are illustrated in Suppl. Tab. 8. Although the likelihood ratio test (LRT) formally indicates a significant improvement by including the additional correlation parameter, the fixed and random estimates as well as the standard errors for intercept and slope are almost identical for both models. This substantiates our decision to use the simpler model, i.e. neglecting autocorrelation, because the aim of our linear regression modelling was to estimate the slope parameter as a proxy describing the individual BCR::ABL1^IS^ trend.

*Risk classification based on the dynamics of 3 months under TKI dose de-escalation*

In our DESTINY cohort 10 out of 107 patients experienced early recurrences during TKI dose de-escalation. For these patients, prediction with respect to TFR was not possible, as they never reached TKI discontinuation. Motivated by this, we investigated whether molecular recurrence prediction is already feasible after the 3-month visit of TKI dose de-escalation. In clinical practice, the time from trial entry to the 3-month visit varies around 3 months; thus, we set the threshold at 3.5 months, still referring to it as “3 months”. We described the time courses of the log_10_[BCR::ABL1^IS^] within the first 3 months of TKI dose reduction by a linear regression model, as outlined above. Since we required at least 3 detectable observations per patient, the eligible cohort decreased to n = 91 patients, with a median of 3 samples per patient (IQR = [3; 4], range = [1; 5]).

To classify these patients into the three risk groups, we applied ROC analysis (as described above) and obtained cutoff values of –3.052 for the intercept and 0.207 for the slope, resulting in 16 low-risk (17.6%), 11 high-risk (12.1%), and 64 unclear-risk (70.3%) patients. For patients classified into the low- or high-risk groups (n = 27), the classification characteristics were: classification error = 22.2% (95% CI: [6.5; 37.9]), PPV = 100%, and NPV = 62.5% (95% CI: [38.8; 86.2]).

Finally, we estimated the molecular recurrence-free survival (MRFS) probabilities after 3 months of TKI dose de-escalation using a Kaplan–Meier estimator, as described above. Two patients with recurrences before month 3 were excluded (one unclear-risk, one high-risk).

These results suggest that prediction of recurrence might be feasible during TKI dose de-escalation (Suppl. Fig. 9). However, the presented cohort does not allow for robust classification and further independent cohorts are needed to determine an optimal time point for earlier prediction.

*Cross-validation of the cut-off values for intercept and slope based on the dynamics of 3-months under TKI dose de-escalation*

To evaluate the corresponding cut-off values for the intercept (-3.052) and slope (0.207) obtained from the ROC analysis in this setting, we again applied the cross-validation (CV) methods LOOCV, leave-10%-out CV, and 5-fold CV. Using the original classification method, 64 patients (70.3%) were classified as unclear-risk, and 27 patients (22.2%) were misclassified. The CV analyses (Suppl. Tab. 9) provided robust estimates of the cutoff values; however, the overall proportion of patients classified as unclear-risk and the misclassification rate were relatively high compared with classification based on the 12-month dynamics under TKI dose de-escalation. For reliable classification after 3 months (i.e. after 3 visits), robust monitoring, and precise data are essential to obtain stable estimates of the dynamics.

**Supplemental Figures**

**
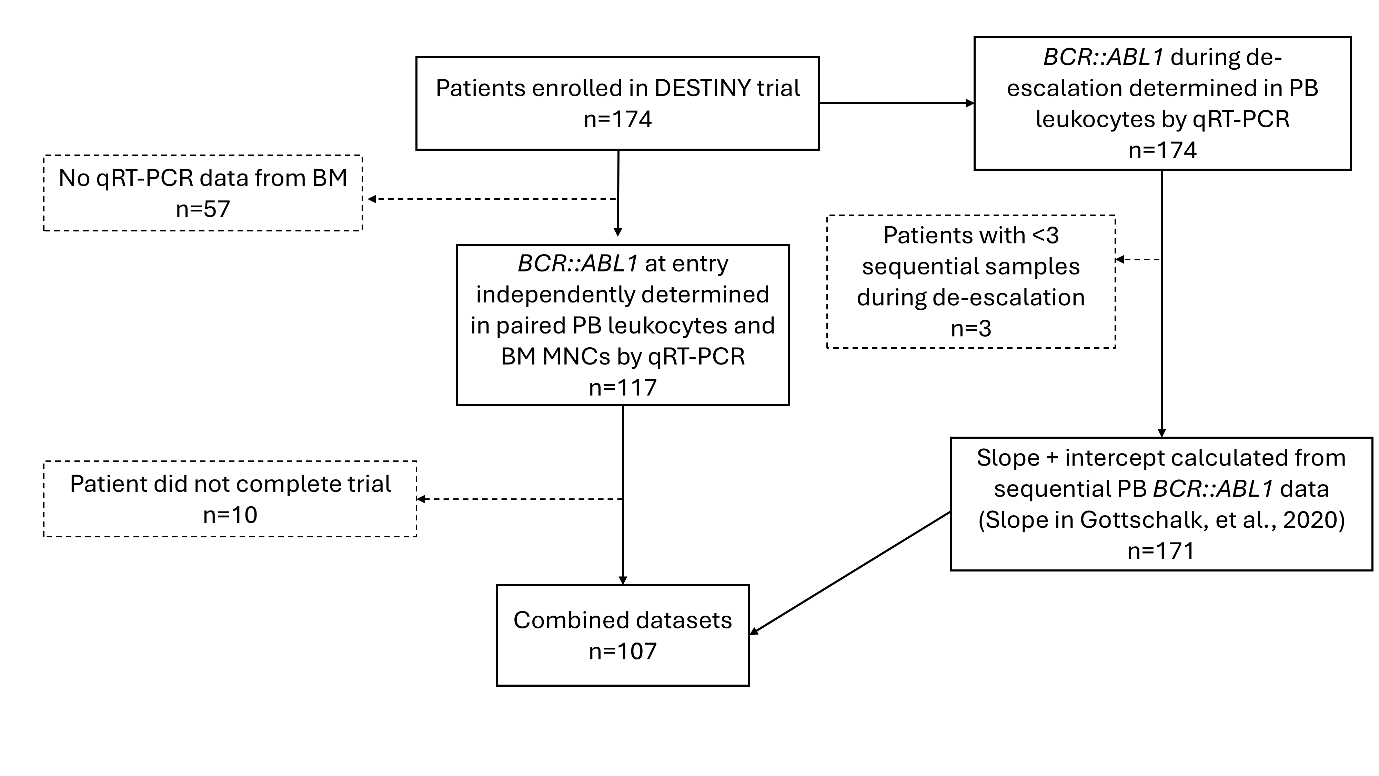
Supplemental Figure 1. Sample selection process.** A subset of BM MNC samples from DESTINY trial participants were randomly selected for BCR::ABL1^IS^ assessment. Samples were later excluded if qRT-PCR was unsuccessful or if there was no available data from paired PB total leukocyte samples. Clinical BCR::ABL1^IS^ transcript data from the PB leukocytes of DESTINY trial participants (n=174) were available (as published in [S3]) at the trial entry point (immediately prior to de-escalation) and sequentially following this period; 3 patients had below the minimum requirement of 3 measurements for the regression and were excluded. The n=10 patients did not complete the study due to reasons other than molecular recurrence, including trial protocol violation, withdrawal of consent or unrelated mortality.

BM; bone marrow, PB; peripheral blood, MNC; mononuclear cell, TFR; treatment-free remission.

A B

C D

**Supplemental Figure 2: Examples of linear regression-based characterization of BCR::ABL1^IS^ dynamics during TKI dose de-escalation according to risk groups.** Shown are representative examples of BCR::ABL1^IS^ dynamics, classified as *low* (blue), *high* (red) or *unclear* (turquoise) risk, together with their slope and intercept (estimated by linear regression for *molecular response* levels in peripheral blood (PB) leukocytes during dose de-escalation). Additionally, BCR::ABL1^IS^*,* measured at a single time point immediately prior to TKI dose de-escalation in BM MNCs or PB leukocytes is shown by dark blue and orange dots, respectively. **A:** Example patient with *low*-risk classification, i.e. intercept < -2.8 and slope < 0.035 (blue dot and line). **B:** Example patient with *high*-risk, i.e. high intercept > -2.8 and high slope > 0.035 (red solid line). **C**/**D:** Example patients with *unclear*-risk (intercept + slope: turquoise dot and line): **C:** intercept > -2.8 and slope < 0.035; **D:** intercept < -2.8 and slope > 0.035. BCR::ABL1^IS^ before TKI dose de-escalation are shown as deep blue (BM) and salmon (PB) dots; levels in PB after TKI dose de-escalation are shown as black dots. Dashed red line: time point of recurrence, if applicable.

BM: bone marrow, PB: peripheral blood, TKI: tyrosine kinase inhibitor, MMR: major molecular response, DMR: deep molecular response, MNCs: mononuclear cells.

**
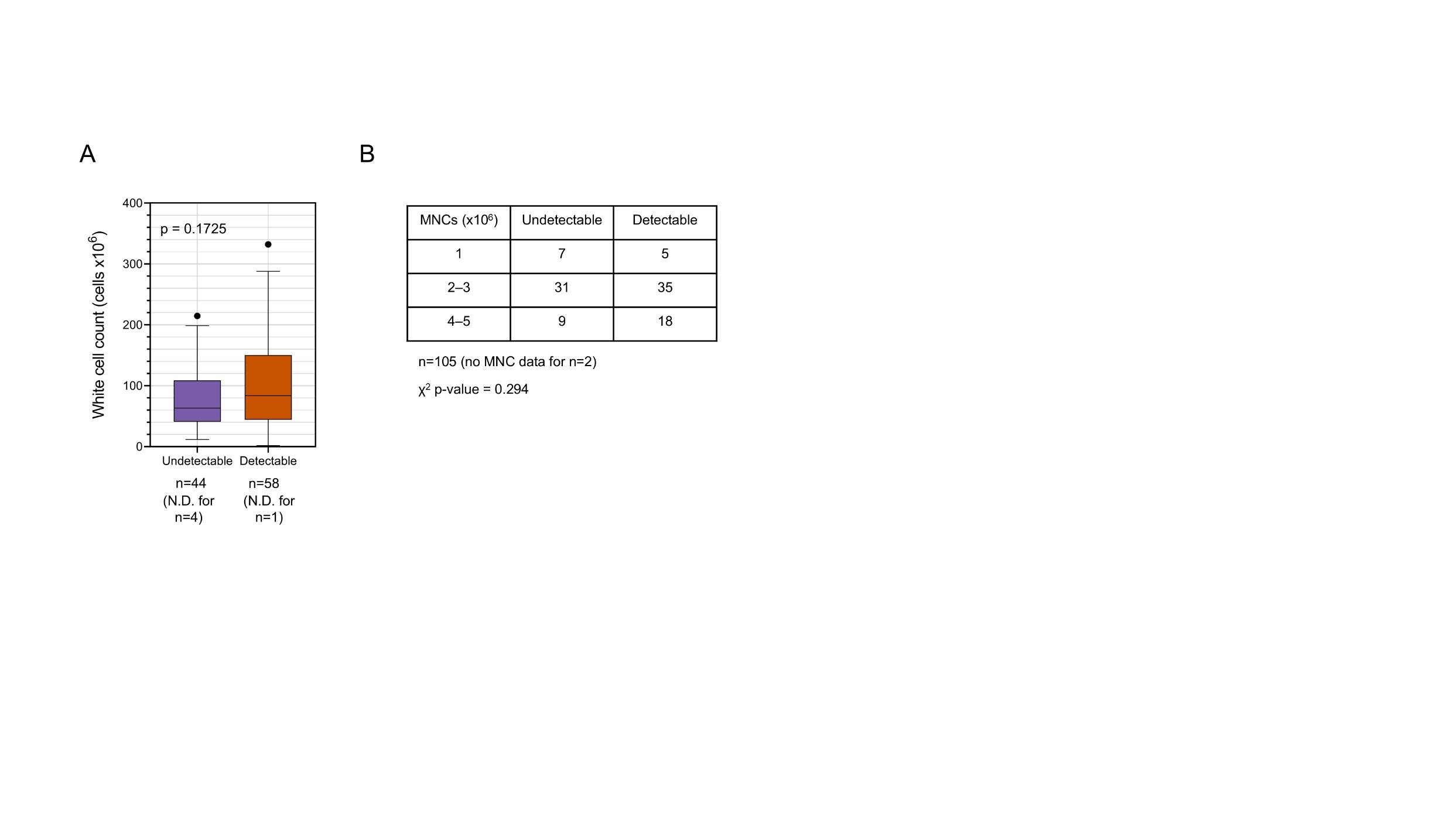
Supplemental Figure 3. Relationship of white blood cell and mononuclear cell counts with BCR:ABL1 detectability.** Neither the total white cell count (adjusted for aspirate volume) of BM aspirates nor the cell count of isolated BM MNCs were associated with BCR::ABL1 detectability by RT-qPCR at a significance level of 5%. The subset of BM MNC samples from DESTINY trial participants that were selected for BCR::ABL1^IS^ assessment were classified as undetectable (BCR::ABL1^IS^ $\leq$ 0.0001%) or detectable (BCR::ABL1^IS^ $>$ 0.0001%). **A:** The white cell counts recorded in the BM aspirate immediately prior to MNC isolation are separated by detectability classification; total white cell count was calculated by adjusting the cell density by recorded aspirate volume. A Mann–Whitney U-test was used to statistically test for a difference between the median of each group. **B:** BM MNC samples are grouped by the number of MNCs used as input for RNA isolation, cDNA preparation and RT-qPCR per sample and by detectability. MNC numbers were rounded to the nearest multiple of 10^6^ cells per sample and aggregated in three groups (1, 2-3, 4-5 x 10^6^ cells). To test for an association between detectability status (columns) and MNC numbers (lines) a χ^2^ test was applied.

BM; bone marrow, MNC; mononuclear cell, N.D.; no data.

A B

**Supplemental Figure 4:**  **BCR::ABL1^IS^ levels immediately prior to TKI dose de-escalation were highest in patients who subsequently relapsed during the de-escalation phase in both BM and PB.** Box plots, showing median (black line) and interquartile range (box), for BCR::ABL1^IS^ as measured in BM MNCs **(A)** and PB leukocytes **(B)**, separated for the three outcome groups: recurrence during TKI de-escalation, i.e., study months 0-12 (red); recurrence during TKI cessation, i.e. study months 13–36 (turquoise); and TFR (no recurrence by 36 study months, blue). P-value refers to testing the statistical trends (i.e., decreasing BCR::ABL1^IS^) using a Kruskal–Wallis test, i.e. testing for equality of all three groups.

BM: bone marrow, PB: peripheral blood, TKI: tyrosine kinase inhibitor, TFR: treatment-free remission, ND: not detectable, MMR: major molecular response, DMR: deep molecular response, MNC: mononuclear cells.

A B

C D

**Supplemental Figure 5: The PB-derived intercept value is more predictive of TFR outcome than single PB leukocyte or BM MNC BCR::ABL1^IS^ measurements, based on univariate logistic regression.** Recurrence probabilities estimated by univariate logistic regression analyses (solid black line, dashed black lines: 95% CI) for the prediction of TFR. **A/B:** single time point immediately prior to TKI dose de-escalation; **A:** BM MNCs, **B:** PB leukocytes**. C/D:** linear regression results, based on sequential PB levels during de-escalation. **C:** intercept, **D:** slope. Dots are referring to individual intercept (left) and slope (right) values of patients who showed TFR (blue) or recurrence (red). Estimated ORs and associated 95% CI describe the increase in the chance of losing TFR if BM, PB or intercept value is increasing by one unit and if slope is increasing by 0.01 unit.

BM: bone marrow, PB: peripheral blood, MNC: mononuclear cells, TKI: tyrosine kinase inhibitor, TFR: treatment-free remission, OR: odds ratio, CI: confidence interval.

**A**

**B**

**Supplemental Figure 6: Classification cut-off determination by ROC analysis.** The intercept (**A**) and slope (**B**) values, determined by linear regression of sequential PB BCR::ABL1^IS^ measurements during TKI dose de-escalation, were evaluated for their ability to predict TFR success using cut-off–specific sensitivities and specificities derived from receiver operating characteristic (ROC) analysis with associated 95% confidence intervals (turquoise band, left). Sensitivity and specificity are defined as: 𝑠𝑒𝑛𝑠 = 𝑡𝑝/(𝑡𝑝 + 𝑓𝑛) and 𝑠𝑝𝑒𝑐 = 𝑡𝑛/(𝑡𝑛 + 𝑓𝑝), with 𝑡𝑝 = true positive, 𝑡𝑛 = true negative, 𝑓𝑝 = false positive and 𝑓𝑛 = false negative. The optimal cut-off (highlighted in the ROC curve, left, and shown in the histogram, right) was determined using the maximum Youden Index: 𝐽 = 𝑠𝑒𝑛𝑠 + 𝑠𝑝𝑒𝑐 − 1.

PB: peripheral blood, TKI: tyrosine kinase inhibitor, TFR: treatment-free remission, ROC: receiver operating characteristic.

**A B**

**Supplemental Figure 7. Risk classification based on single timepoint measurement before TKI de-escalation.** Outcomes (TFR vs. recurrence) at 24 months post TKI stop are shown for participants classified into *low* and *high*-risk prediction groups, based on a previously determined cut-off (BM: -2.27, PB: -2.52) as measured in BM MNCs (**A**) or PB leukocytes (**B**). Statistics associated with the predictive models are (**A**) BM: OR = 8.47 with 95% CI = [2.57; 27.92], PPV = 78,9% with 95% CI = [60.6%; 97.3%], NPV = 69.3% with 95% CI = [59.7%; 79.0%], misclassification: 29.0% with 95% CI [20.4; 37.6] and (**B**) PB: OR = 5.63 with 95% CI = [2.08; 15.27], PPV = 70,8% with 95% CI = [52.6%; 89.0%], NPV = 69.9% with 95% CI = [60.0%; 79.7%], misclassification: 29.9% with 95% CI [21.2: 38.6].

BM: bone marrow, PB: peripheral blood, MNCs: mononuclear cells, TKI: tyrosine kinase inhibitor, TFR: treatment-free remission, OR: odds ratio, CI: confidence interval, PPV: positive predictive value, NPV: negative predictive value.

| **** | **** |
| --- | --- |
| **** | **** |
| **** | **** |
| **** | **** |
| **** | **** |

**Supplemental Figure 8. BCR::ABL1^IS^** **dynamics of all patients with recurrence within the tyrosine kinase inhibitor dose de-escalation phase.** Shown are the BCR::ABL1^IS^ dynamics for patients with recurrence before TKI stopping (dynamics from all other patients can be obtained from the authors upon request), classified as *high-* (i.e. high intercept > -2.8 and high slope > 0.035, red) or *unclear*- (i.e. intercept < -2.8 and slope > 0.035, turquoise) risk, together with slope and intercept. BCR::ABL1^IS^ levels before TKI dose de-escalation are shown as deep blue (BM MNC) and salmon (PB leukocytes) dots; BCR::ABL1^IS^ levels in PB after TKI dose de-escalation are shown as black dots. Vertical dashed red line: Time point of recurrence. The slope and intercept estimates have been obtained from measurements of the first three months after TKI dose de-escalation, only (dotted increasing lines and same diamond, with cut-offs for high intercept > –3.052 and high slope > 0.207) and from all measurements after TKI dose de-escalation (solid increasing lines and same colored dot, with cut-offs for high intercept > -2.8 and high slope > 0.035). The uncertainty in risk classification (i.e., *unclear*-risk as shown by turquoise slope and intercept) results from a rather low intercept (below MR5), such that even the large slope does not allow a *high*-risk classification according to the rules described.

BM: bone marrow, PB: peripheral blood, MNC: mononuclear cells, TKI: tyrosine kinase inhibitor, MR5: molecular response level 5.

**Supplemental Figure 9: Risk of relapse following 3 months under TKI dose de-escalation. A:** Proportion of patients in TFR or with recurrence evaluated between months 3 of dose de-escalation and the end of the 24-month follow-up period (after TKI stop) across the three risk groups based on classification using intercept and slope from a 3-month TKI dose de-escalation period. Absolute patient numbers given below the bars. **B:** Kaplan–Meier curve for molecular recurrence-free survival (MRFS) (shaded areas: 95% confidence intervals), stratified according to the three risk classification groups; low, high and unclear. The dashed line represents the end of the de-escalation phase (12 months). The trial endpoint was set to 36 months, i.e., 24-month follow-up post-TKI cessation. Thus, patients were censored at last seen date before or at the end of trial.

| **** | **** |
| --- | --- |
| **** |  |
| **** | **** |

**Supplemental Figure 10. BCR::ABL1^IS^** **dynamics of all patients who experienced recurrence after the DESTINY trial endpoint, i.e., more than 24 months post TKI stop.** Explanation of symbols: see Suppl. Fig. 8. Additionally, undetectable BCR::ABL1^IS^ levels are shown as triangles. Measurements after 24 months post TKI stop are missing but the time points of recurrence are available (dashed red line). Risk classification calculated according to the assumption that these patients did stay in TFR, as they did within the 24-month post-TKI cessation follow-up period. These cases illustrate the dependence of the risk classification (including the cut-off choice) on the follow-up time that is considered to evaluate TFR / loss of TFR. However, BM MNC and PB leukocyte molecular response levels prior TKI dose de-escalation (light blue and purple dots) are low (BM: median [IQR] = -2.8 [-4; -2.6], PB: -3.2 [-4; -2.9]) and comparable to those of other TFR patients (BM: median [IQR] = -4 [-4; -2.59]; PB: -2.98 [-3.44; -2.81]).

BM: bone marrow, PB: peripheral blood, MNC: mononuclear cells, TKI: tyrosine kinase inhibitor, TFR: treatment-free remission, IQR: interquartile range.

**Supplemental Figure 11. Sample size effect on the precision of the estimates.** Shown are the associations between sample size per patient and the estimated standard errors for the intercept and slope obtained from the linear regression of log_10_[BCR::ABL1^IS^] during the 12-month TKI dose de-escalation period. For correlation analysis, the Spearman’s correlation coefficient (R) is shown with the associated p-value.

**Supplemental Tables**

**Supplemental Table 1. Demographics of DESTINY trial participants.** Demographic data from the subset of trial participants from whom samples were used in this study (n=107). Percentages are within each outcome group.

DAS: dasatinib, DMR: deep molecular response, IM: imatinib, MMR: major molecular response, NIL: nilotinib, TFR: treatment-free remission, TKI: tyrosine kinase inhibitor.

| Assay | Primer/Probe Name | Gene [Exon] | Sequence (5’-3’) | Final Concentrations |
| --- | --- | --- | --- | --- |
| *BCR::ABL1* | ENF501 | *BCR [13]* | TCCGCTGACCATCAAYAAGGA | 300nM |
|  | ENR561 | *ABL1 [2]* | CACTCAGACCCTGAGGCTCAA | 300nM |
|  | FAM-ENP541-MGB | *ABL1 [2]* | FAM-CCCTTCAGCGGCCAGT-MGB | 100nM |
| *ABL1* | ENF1003 | *ABL1 [2]* | TGGAGATAACACTCTAAGCATAACTAAAGGT | 150nM |
|  | ENR1063 | *ABL1 [3]* | GATGTAGTTGCTTGGGACCCA | 150nM |
|  | VIC-ABL1043-MGB | *ABL1 [3]* | VIC-CATTTTTGGTTTGGGCTTC-MGB | 200nM |

**Supplemental Table 2. Primer and Probe Sequences and Final Concentrations.** The primers, probes and final concentrations utilised in the duplex RT-qPCR reactions are those published [S5].

| **model** | **Coefficient** | **Estimate** | **Std. Error** | **p-value** |
| --- | --- | --- | --- | --- |
| A) BM | (offset) | 1.3601 | 0.7257 |  |
|  | BM | 0.5893 | 0.2305 | 0.0106 |
|  | residual std. deviation σ | 136.19 |  |  |
| B) PB | (offset) | 2.3548 | 1.0902 |  |
|  | PB | 0.9561 | 0.3705 | 0.0099 |
|  | residual std. deviation σ | 136.01 |  |  |
| C) intercept | (offset) | 4.2205 | 1.4643 |  |
|  | intercept | 1.6617 | 0.5178 | 0.0013 |
|  | residual std. deviation σ | 131.48 |  |  |
| D) slope | (offset) | -1.46448 | 0.30903 |  |
|  | slope | 0.33360 | 0.07878 | <0.001 |
|  | residual std. deviation σ | 94.16 |  |  |
| E) intercept + slope | (offset) | 5.9520 | 2.1859 |  |
|  | intercept | 2.6253 | 0.7830 | <0.001 |
|  | slope | 0.4163 | 0.1023 | <0.001 |
|  | residual std. deviation σ | 80.27 |  |  |
| F) intercept + slope + BM | (offset) | 5.97966 | 2.34426 |  |
|  | BM | 0.01107 | 0.33766 | 0.9738 |
|  | intercept | 2.62252 | 0.78728 | <0.001 |
|  | slope | 0.41608 | 0.10244 | <0.001 |
|  | residual std. deviation σ | 80.27 |  |  |
| G) intercept + slope + PB | (offset) | 5.7524 | 2.2257 |  |
|  | PB | -0.5444 | 0.5929 | 0.3585 |
|  | intercept | 3.1388 | 0.9853 | 0.0014 |
|  | slope | 0.4391 | 0.1098 | <0.001 |
|  | residual std. deviation σ | 79.44 |  |  |

**Supplemental Table 3. Model parameters from all logistic regression models applied.** Coefficient estimates and corresponding standard deviations of logistic regression models for TFR success together with p-value of the Wald significance test for predictor effects: **A)** Univariate model with single BCR::ABL1^IS^ from bone marrow (BM) mononuclear cells (MNCs) taken immediately prior to de-escalation as predictor, **B)** univariate model with single BCR::ABL1^IS^ from peripheral blood (PB) leukocytes taken immediately prior to de-escalation as predictor, **C)** univariate model with intercept (estimated by linear regression of multiple BCR::ABL1^IS^ from PB leukocytes during de-escalation) as a predictor, **D)** univariate model with slope (estimated by linear regression of multiple BCR::ABL1^IS^ from PB leukocytes during de-escalation) as predictor, **E)** bivariate model with slope and intercept (estimated by linear regression of multiple BCR::ABL1^IS^ from PB leukocytes during de-escalation) as predictors, **F)** combination of model A) and D) and **G)** combination of model B) and D). The parameters BM MNCs, PB leukocytes, slope and intercept are reported in terms of BCR::ABL1^IS^ level. The variable slope was multiplied by 100 for better interpretation and robust fitting.

BM: bone marrow, PB: peripheral blood, MNCs: mononuclear cells, TFR: treatment-free remission.

| **model** | **AIC** | **p-value** (compared to reference model) |
| --- | --- | --- |
| **Univariate models** | | |
| A) BM | 140.19 |  |
| B) PB | 140.01 |  |
| C) intercept | 135.48 | <0.001 |
| D) slope | 98.16 | <0.001 |
| **Multivariate models** | | |
| **E) intercept + slope (reference)** | **86.27** |  |
| F) intercept + slope + BM | 88.27 | 0.9738 |
| G) intercept + slope + PB | 87.44 | 0.3633 |

**Supplemental Table 4. Comparison of logistic regression models.** AIC of all logistic regression models for TFR success of Suppl. Table 3. p-values refer to pairwise model comparisons with model E (intercept + slope) as reference model using likelihood ratio test. Comparison of all univariate models, does not show any significant differences. Model E (intercept + slope), which is significantly more appropriate than the univariate models C (intercept) and D (slope) shows lowest AIC among all fitted models. Extending model E by either BM MNC or PB leukocyte values measured immediately prior to TKI dose de-escalation does not result in a significant change to the model fit.

BM: bone marrow, PB: peripheral blood, MNC: mononuclear cell, TFR: treatment-free remission, TKI: tyrosine kinase inhibitor, AIC: Akaike information criterion.

| **parameter** | **cut-off value** | **number of *high-risk* patients**  **n (%)** | **classification error [95%CI]** | **PPV**  **% [95%CI]** | **NPV**  **% [95%CI]** |
| --- | --- | --- | --- | --- | --- |
| BM | -2.27 | 19 (17.8) | 29.0  [20.4; 37.6] | 78.9,  [60.6; 97.3] | 69.3  [59.7; 79.0] |
| PB | -2.52 | 24 (22.4) | 29.9  [21.2; 38.6] | 70,8  [52.6; 89.0 | 69.9  [60.0; 79.7] |
| intercept | -2.8 | 38 (35.5) | 26.2  [17.8; 34.5] | 68.4  [53.6, 83,2] | 76.8  [66.9; 86.8] |
| slope | 0.035 | 35 (32.7) | 20.2  [12.6; 27.7] | 77.8%  [64.2; 91.4] | 80.8  [71.8; 89.9] |
| slope  +  Intercept* | 0.035  -2.8 | 20 (27.0) | 10.8  [3.7; 17.9] | 100 | 85.2  [75.7; 94.7] |

**low-* and *high*-risk groups only (n=74)

**Supplemental Table 5:** **Characteristics of the cut-off values of the different parameters and the reference model (n=107).** Given are the cut-off values based on the parameters BM MNCs, PB leukocytes, intercept and slope (alone and jointly), with the resulting number of high-risk patients. Classification error, PPV and NPV are reported with respect to the outcomes TFR and molecular recurrence after trial entry.

BM: bone marrow, PB: peripheral blood, MNC: mononuclear cells, PPV: positive predictive value, NPV: negative predictive value.

|  | **no recurrence** | | | **recurrence** | | | **OR**  **[95%CI]** |
| --- | --- | --- | --- | --- | --- | --- | --- |
|  | Low risk | High risk | Unclear risk | Low risk | High risk | Unclear risk |  |
| BM | 61 | 4 |  | 27 | 15 |  | 8.47  [2.57; 27.92] |
| PB | 58 | 7 |  | 25 | 17 |  | 5.63  [2.08; 15.27] |
| intercept | 53 | 12 |  | 16 | 26 |  | 7.18  [2.97; 17.36] |
| slope | 58 | 7 |  | 14 | 28 |  | 16.57  [6.02; 45.65] |
| slope + intercept | 46 | 0 | 19 | 8 | 20 | 14 | - |

**Supplemental Table 6: Contingency table showing the number of patients classified for recurrence status after trial entry and the particular parameter(s) (n=107).** BM: bone marrow, PB: peripheral blood, TFR; treatment-free remission, OR: odds ratio, CI: confidence interval.

|  | **repeats** | **Estimation in training set** | | | **Classification in test set** | | |
| --- | --- | --- | --- | --- | --- | --- | --- |
|  |  | Nr. of samples per set | Median  intercept  [IQR] | Median slope  [IQR] | Nr. of samples per set | Median nr. of unclear-risk patients  (median %) | Median nr. of mis-classification*  (median %) |
| **CV method** | | | | | | | |
| **LOOCV** | 107 | 106 | -2.800  [-2.800;  -2.800] | 0.035 [0.035; 0.035] | 1 | 34 (31.8) | 8 (11.0) |
| **Leave 10% out - CV** | 1000 | 96 | -2.800  [-2.800;  -2.800] | 0.035 [0.035; 0.035] | 11 | 4 (36.4) | 1 (12.5) |
| **5-fold CV** | 5 | 86 | -2.800  [-2.800;  -2.800] | 0.035 [0.035; 0.035] | 21 | 7 (33.3) | 2 (14.3) |
| **Original classification (identical training and test set)** | | | | | | | |
|  |  | 107 | -2.800 | 0.035 | 107 | 33 (30.8) | 8 (10.8) |

*of clearly classified patients

**Supplemental Table 7: Cross-validation results for 12 months-based classification.** Cross-validation of intercept and slope cut-off values obtained from the ROC analysis, based on time course data from 12-month TKI dose de-escalation (n = 107). IQR, interquartile range; CV, cross-validation; LLOCV, leave-one-out CV.

| **model** | **Coefficient** | **Estimate** | **Std. Error** | **Wald-test**  **p-value** | **AIC** | **LRT**  **p-value** |
| --- | --- | --- | --- | --- | --- | --- |
| **A) Basic (reference) model** | | | | | | |
| Fix effects | intercept | -2.7841 | 0.0453 | <0.001 | 1050.53 |  |
|  | slope | 0.0352 | 0.0069 | <0.001 |  |  |
| Random effects | intercept std. deviation | 0.4278 |  |  |  |  |
|  | slope std. deviation | 0.0637 |  |  |  |  |
|  | residual std. deviation σ | 0.3056 |  |  |  |  |
| **B) Extended model with autoregression** | | | | | | |
| Fix effects | intercept | -2.7841 | 0.0458 | <0.001 | 1016.41 | <0.001 |
|  | slope | 0.0327 | 0.0063 | <0.001 |  |  |
| Random effects | intercept std. deviation | 0.4130 |  |  |  |  |
|  | slope std. deviation | 0.0545 |  |  |  |  |
|  | residual std. deviation σ | 0.3222 |  |  |  |  |
|  | autocorrelation parameter $\phi$ | 0.2039 |  |  |  |  |

**Supplemental Table 8: Model parameters from population-based linear mixed-effects models. Model A)** Coefficient estimates and corresponding standard deviations of linear mixed effects models for log_10_[BCR::ABL1^IS^] values during the 12-month TKI dose de-escalation period are shown, together with p-value of the Wald significance test for predictor effects**.** **Model B)** As in model A), but extended by including a continuous autoregressive process of order 1 with the corresponding autocorrelation parameter $\phi$. For model comparison, the Akaike Information Criterion (AIC) and the p-value from the likelihood ratio test (LRT) are reported.

|  | **repeats** | **Estimation in training set** | | | **Classification in test set** | | |
| --- | --- | --- | --- | --- | --- | --- | --- |
|  |  | Nr. of samples per set | Nr. of samples per set | Nr. of samples per set | Nr. of samples per set | Median nr. of unclear-risk patients  (median %) | Median nr. of mis-classification*  (median %) |
| **CV method** | | | | | | | |
| **LOOCV** | 91 | 90 | -3.052  [-3.052;  -3.052] | 0.207 [0.207; 0.207] | 1 | 62 (68.1) | 14 (48.3) |
| **Leave 10% out - CV** | 1000 | 81 | 3.052  [-3.052;  -3.052] | 0.207 [0.055; 0.207] | 10 | 7 (70.0) | 1 (33.3) |
| **5-fold CV** | 5 | 73 | -3.052  [-3.052;  -3.052] | 0.078 [0.078; 0.207] | 18 | 12 (66.7) | 2 (33.3) |
| **Original classification (identical training and test set)** | | | | | | | |
|  |  | 91 | -3.052 | 0.207 | 91 | 64 (70.3) | 27 (22.2) |

*of clearly classified patients

**Supplemental Table 9: Cross-validation results for 3 months-based classification.** Cross-validation of intercept and slope cut-off values obtained from the ROC analysis, based on time course data from 12-month TKI dose de-escalation (n = 107). IQR, interquartile range; CV, cross-validation; LLOCV, leave-one-out CV.

**Supplemental references**

S1. Clark RE, Polydoros F, Apperley JF, Milojkovic D, Pocock C, Smith G, et al. De-escalation of tyrosine kinase inhibitor dose in patients with chronic myeloid leukaemia with stable major molecular response (DESTINY): an interim analysis of a non-randomised, phase 2 trial. Lancet Haematol. 2017;4(7):e310-e6. doi: 10.1016/s2352-3026(17)30066-2.

S2. Clark RE, Polydoros F, Apperley JF, Milojkovic D, Rothwell K, Pocock C, et al. De-escalation of tyrosine kinase inhibitor therapy before complete treatment discontinuation in patients with chronic myeloid leukaemia (DESTINY): a non-randomised, phase 2 trial. Lancet Haematol. 2019;6(7):e375-e83. doi: 10.1016/s2352-3026(19)30094-8.

S3. Gottschalk A, Glauche I, Cicconi S, Clark RE, Roeder I. Molecular monitoring during dose reduction predicts recurrence after TKI cessation in CML. Blood. 2020;135(10):766-9. doi: 10.1182/blood.2019003395.

S4. Youden WJ. Index for rating diagnostic tests. Cancer. 1950;3(1):32-5. doi: 10.1002/1097-0142(1950)3:1<32::aid-cncr2820030106>3.0.co;2-3.

S5. Gerrard G, Mudge K, Foskett P, Stevens D, Alikian M, White HE, et al. Fast-mode duplex qPCR for BCR-ABL1 molecular monitoring: Innovation, automation, and harmonization. American Journal of Hematology. 2012;87(7):717-20. doi: 10.1002/ajh.23212.
